# Outage Performance Improvement by Selected User in D2D Transmission and Implementation of Cognitive Radio-Assisted NOMA

**DOI:** 10.3390/s19224840

**Published:** 2019-11-06

**Authors:** Dinh-Thuan Do, Minh-Sang Van Nguyen, Byung Moo Lee

**Affiliations:** 1 Wireless Communications Research Group, Faculty of Electrical and Electronics Engineering, Ton Duc Thang University, Ho Chi Minh City 700000, Vietnam; 2 Faculty of Electronics Technology, Industrial University of Ho Chi Minh City (IUH), Ho Chi Minh City 700000, Vietnam; nguyenvanminhsang@iuh.edu.vn; 3 School of Intelligent Mechatronics Engineering, Sejong University, Seoul 05006, Korea

**Keywords:** cognitive radio, NOMA, outage probability, D2D

## Abstract

In this paper, we investigate the outage performance in secondary network of cognitive radio (CR) employing non-orthogonal multiple access (NOMA) wireless networks over Rayleigh fading channels. The considered system model adopts device-to-device (D2D) transmission together with traditional communication to form a new system model, namely CR-D2DNOMA network. The specific user is selected from multiple D2D-Tx users (D2Ds) to communicate with far NOMA users to form qualified D2D connection with assistance of the Relay user (RU). The main metric in such CR-D2DNOMA network needs to be considered and we particularly introduce the closed-form expressions for outage probability in the secondary network where it is designed to serve two far NOMA users. The perfect Successive Interference Cancellation (SIC) and imperfect SIC can be further examined at the second NOMA user who detects signal based on the ability of SIC. The results show the positive impact of increasing the fading parameters on the system performance. More importantly, numerical results are provided to verify the correctness of our derivations. Additionally, the effects of asymptotic expressions on insights evaluation are also further analyzed.

## 1. Introduction

To improve current statistic spectrum use, cognitive radio (CR) is proposed as a spectrum-sharing technique in which a primary user (PU) permits a secondary user (SU) to occupy idle spectrum assigned to such PU. However, the normal communications of the PU must satisfy since non-existence of interference between the SUs and the PUs. Regarding capability of spectrum sensing, a binary detection problem is way to help the SU sense the idle spectrum as in [1,2,3]. In the traditional system, the idle channel is allocated to the SU when the absence of the PU is detected. In the other hand, SU is not able to operate due to lack of channel in case of full occupation of PU’s channels. By comparing between a prior known threshold and the accumulated energy statistic of the PU signal, such energy detection scheme is widely employed to detect the PU. If the energy statistic is less than the threshold, the PU is detected to be absent [4,5,6,7]. detection probability and false alarm probability are two factors affecting the performance of energy detection. highly reliable detection of the idle channel can be achieved as low false alarm probability happened, while highly accurate detection on the presence of the PU results from high detection probability. To improve detection probability and decrease false alarm probability, the detection performance depends on the sensing time [8,9]. Such technique can be implemented to design 5G wireless such as recent results reported in [10]. The authors in [11] presented spectrum access scheme, namely listen-before-talk, in which sensing slot and transmission slot are both arranged in the frame and the transmission slot contains the SU’s signal, then the idle channel was identified in the sensing slot.

To provide reliable communication, the authors in [12,13] reported advantages of relaying network in terms of outage performance. By permitting transmission in two hop, system improvement can be achieved since it combines relaying scheme with non-orthogonal multiple access (NOMA). Regarding improvement of spectrum efficiency, same advantages found in CR, NOMA is promising spectrum enhancement technique. Cooperative NOMA with advantages in system performance is introduced in recent works [14,15,16]. To manage interference in these NOMA networks, successive interference cancellation (SIC) is required. Both primary user and secondary user can be served in the same time as combining NOMA and CR and such hybrid scheme does not harm on performance of primary users [17]. Furthermore, the potential to realize ubiquitous connectivity for future networks would be extra benefit from the combination of NOMA and CR. In the context of CR in NOMA systems, a secondary user who possess a strong channel condition, and it is squeezed into the spectrum involved in a primary user using a poor channel condition [18]. As a result, the interference of secondary user can be limited when implementing NOMA in a cognitive network. As report from [18], it can be remained the quality of service (QoS) for users possessing poor channel conditions in such CR-NOMA because the transmit power allocated to other users is limited to satisfy power constraint from cognitive radio deployment. As main numerical result, this paper indicated a different behavior achieved from CR-NOMA can be seen in comparison with conventional NOMA due to this constraint [18].

From recent results reported in [19,20,21], capable of further improving the spectrum efficiency is main benefit from the combination of CR with NOMA. The second reason regarding popularity of, implementation on spectrum-sharing technique due to low implementational complexity. Main conclusion from in [19,20,21] indicated that NOMA can outperform traditional orthogonal multiple access in underlay CR networks by careful selections in terms of power allocation coefficients and target data rates of users. For example, new closed-form formula of the outage probability is derived and such important metric is used to characterize the performance of the considered network relying D2D transmission [19]. The authors in [20] studied hybrid scheme to achieve benefits from NOMA and OMA in reasonable manner. The second users first perform energy harvesting for spectrum sensing and an overlay and underlay mode are required to information transmission [21]. Furthermore, to maximize the achievable throughput for the CR network, the optimization problem is addressed while it remains constrained condition on the total the minimum rate requirements and transmission power of the secondary users [21]. Based on the available channel state information (CSI), application of NOMA to multicast cognitive radio networks (termed as MCR-NOMA) is investigated in terms of three different secondary user scheduling schemes [22]. To highlight advantage of cooperative, they provided new metric, termed as mutual outage probability [22].

In the other work, to enhance the spectral efficiency, there is existence of both primary users and secondary users in common network relying cooperative NOMA and it can be considered to be a special case of multiple access [23]. The other cooperative NOMA assisted underlay cognitive radio network is considered through approximate expressions for the outage probability and a relay is known as selected secondary user [24]. By assuming that the primary source’s interference is considered to be a constant, two metrics including the outage probability and the ergodic capacity to highlight performance of underlay cognitive radio network using cooperative NOMA [25].

To improve quality of received signal, the authors in [26,27,28,29] examined the multi-relay scenario. In cognitive radio networks, the best secondary user is selected to forward the signal to PU and the farthest SU using NOMA protocol while the base station (BS) transmits superimposed signals to both multiple secondary users and primary user (PU) in the same time [27]. Three secondary user scheduling strategies using multicast network to improve performance of both primary and second systems in terms of the robustness of data transmissions [30].

As an important paradigm in 5G systems, Device-to-device (D2D) communication has been investigate and has drawn lots of papers [31,32,33]. In principle, devices are allowed to communicate directly through cellular channels in D2D transmission. Such D2D scheme not only reduces the workload of the base station (BS) but also enhance spectrum efficiency [34,35,36,37]. In D2D scheme, it can be admitted existence of the device with qualified signal need help of the BS who provides reliability connection while main task of the BS is serving traditional cellular users. This paper first introduces user selection schemes in such CR-D2DNOMA network.

To summarize, the contribution of this paper is as follows:

- Motivated from recent results in [38], we only concern performance of D2D links in secondary network of CR-D2DNOMA system. It is worth noting that normal Relay user (RU) is official served by the base station (BS) in the secondary network. While RU assists D2D transmission, it can receive signal from the BS. This situation makes interference harm operation of D2D link.
- Impact of interference from the primary source (PS) in the primary network on the secondary network’s performance in such D2D assisted CR-NOMA is studied. The performance degradation can be controlled by limiting interference source and total transmit power constraint in CR-NOMA.
- We provide main metric to compare performance between CR-NOMA and CR-OMA. In particular, exact closed-form expressions for the outage probability are provided. As expected result, it provides advantages of these techniques including D2D, NOMA, and CR.
- Selected user is allowed to perform D2D transmission through nearby cellular user which forwards NOMA signals to far D2D users. Therefore, the traditional cognitive radio network can further serve D2D users with improved outage performance while remaining quality for normal cellular users.
- These results can be useful in many practical applications, for example, evaluating the traditional channel model in CR-D2DNOMA network such as Rayleigh fading is resulted from this paper.

The rest of this paper is organized as follows: In Section 2, we illustrate our proposed cooperative relaying-based NOMA scheme. Section 3 analyzes the received signal and then outage performance is evaluated. Asymptotic expressions are further provided to look insight performance. Section 4, we illustrate our proposed cognitive radio network serving D2D connection based on OMA scheme. Numerical and simulation results are presented in Section 5. Section 6 concludes this paper.

## 2. System Model

**Figure 1:**
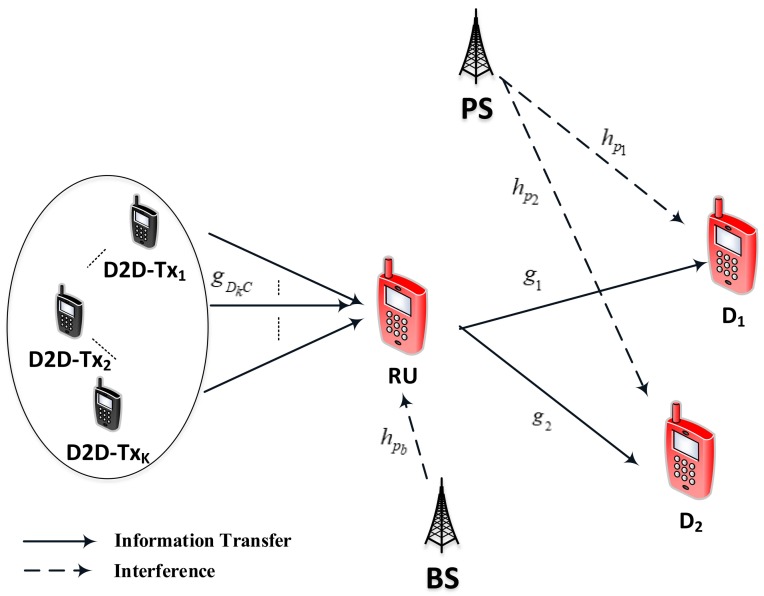
D2D transmission in CR-inspired NOMA.

This Figure 1 illustrates system model to implement a D2D transmission in NOMA-based cognitive radio (CR-D2DNOMA). As two main modes of D2D link, D2D users can communicate directly or through normal cellular user, but this paper considers the second cases. It is worth noting that the selected D2D link need help of nearby RU [39]. This system contains one group containing *K*D2D−Tx users (D2Ds) $\left(D2D-Tx_{k},k=1,\ldots ,K\right)$. These D2D−Tx users in the secondary network of such CR-D2DNOMA intend to communicate with far D2D users via Relay user (RU). In particular, D2D link employing NOMA scheme to serve many destinations. The far D2D users can be received signal via nearby RU without changing allocated power factors at RU. In addition, only selected D2D−Tx can communicate with D2D users under supporting by RU. More specifically, only strong link between D2D−Tx and RU is selected to allow possible D2D connection since large number of D2D−Tx located at different places and it reduces processing load at RU. It is assumed users distributed in a disc with radius *D*, the RU and far users are placed at edge while the BS is in the center. In general, there are two groups, i.e., the first group includes all D2D−Tx, the second group contains D2D users (NOMA users). One pair of D2D users that can use the downlink spectrum resource of the RU. It is assumed that all the D2D−Tx, RU and D2D users are equipped with one single antenna. It is denoted that $g_{D_{k}C}$ is channel in link from $\left(D2D-Tx_{k},k=1,\ldots ,K\right)$ to RU, $h_{pb},h_{p_{1}},h_{p_{2}}$ are channels of links BS−RU, $PS-D_{1}$, $PS-D_{2}$, respectively; $g_{1},g_{2}$ are channels of links $RU-D_{1}$, $RU-D_{2}$. Assume that the complex channel coefficient $h_{u}∼CN\left(0;\lambda_{u}\right),\left(u=\left\{pb,p_{1},p_{2}\right\}\right)$; $g_{D_{k}C}∼CN\left(0;\lambda_{DC}\right)$ and $g_{i}∼CN\left(0;\lambda_{i}\right),\left(i=\left\{1,2\right\}\right)$ with zero mean and variance $\lambda_{u}$, $\lambda_{DC}$ and $\lambda_{i}$, respectively. These channels follow Rayleigh fading model. While $x_{1},x_{2}$ are signals in NOMA scheme, $x_{PS}$ is the PS’s signal and $x_{BS}$ is the BS’s signal. A two-phase transmission framework with time interval of *T* seconds is adopted. The RU need be forwarded signal after performing Decode-and-Forward procedure on received signal from the dedicated D2D−Tx to far NOMA-based D2D users. Such D2D connection need assistance of the RU due to long transmission among pair of D2D users and it occupies two phases. To make analysis simple and without of general, same power allocation factors $\varepsilon_{1},\varepsilon_{2}$ are employed for two transmission phases. In addition, D2D connection is affected by interference from transmit source PS in the primary network of considered CR-D2DNOMA network and it is affected by interference from transmit source BS in the second network.

It is assumed that only user D2D−Tx is permitted to require D2D connection with far NOMA users. $x_{1},x_{2}$ are two signals corresponding to two destinations $D_{1},D_{2}$. In this scenario, the received signal $y_{DC}$ corresponding transmit signal from D2D−Tx at the RU can be expressed by

(1) $$y_{DC}=\sqrt{P_{D2D}}g_{C_{k}B}\left(\sqrt{\varepsilon_{1}}x_{1}+\sqrt{\varepsilon_{2}}x_{2}\right)+\sqrt{P_{BS}}h_{pb}x_{BS}+\omega_{RU},$$

where $P_{D2D}$ and $P_{BS}$ are the transmit powers of selected D2D−Tx and BS, respectively. $\varepsilon_{i}\left(i\in \left\{1,2\right\}\right)$ is the power allocation coefficient for $x_{i}$ with $\varepsilon_{1}+\varepsilon_{2}=1$ and $\varepsilon_{1}>\varepsilon_{2}$, and $\omega_{RU}$ is the AWGN at D2D−Tx to RU.

The signal to interference plus noise ratio (SINR) to decode $x_{1}$ at D2D−Tx to RU is given by

(2) $$\gamma_{D_{k}C,x_{1}}=\frac{\varepsilon_{1}P_{D2D}{\left|g_{D_{k}C}\right|}^{2}}{\varepsilon_{2}P_{D2D}{\left|g_{D_{k}C}\right|}^{2}+P_{BS}{\left|h_{pb}\right|}^{2}+N_{0}}.$$

After SIC, the SINR to decode $x_{2}$ is given by [38]

(3) $$\gamma_{D_{k}C,x_{2}}=\frac{\varepsilon_{2}P_{D2D}{\left|g_{D_{k}C}\right|}^{2}}{P_{BS}{\left|h_{pb}\right|}^{2}+N_{0}}.$$

In the second phase, RU transmits the signal consisting of the decoded and re-encoded symbols to the far D2D users. We call $x_{PS}$ is interference signal from the primary network. The received signal at $D_{i},i=1,2$ is given by

(4) $$y_{D_{1}}=\sqrt{P_{RU}}g_{1}\left(\sqrt{\varepsilon_{1}}x_{1}+\sqrt{\varepsilon_{2}}x_{2}\right)+\sqrt{P_{PS}}h_{p_{1}}x_{PS}+\omega_{D_{1}},$$

(5) $$y_{D_{2}}=\sqrt{P_{RU}}g_{2}\left(\sqrt{\varepsilon_{1}}x_{1}+\sqrt{\varepsilon_{2}}x_{2}\right)+\sqrt{P_{PS}}h_{p_{2}}x_{PS}+\omega_{D_{2}},$$

where $P_{RU}$ and $P_{PS}$ are the transmit powers of selected RU and PS, respectively. $\omega_{D_{i}}$ is the AWGN at $D_{i}$.

The SINR to decode $x_{1}$ at $D_{1}$ is formulated by

(6) $$\gamma_{D_{1},x_{1}}=\frac{\varepsilon_{1}P_{RU}{\left|g_{1}\right|}^{2}}{\varepsilon_{2}P_{RU}{\left|g_{1}\right|}^{2}+P_{PS}{\left|h_{p_{1}}\right|}^{2}+N_{0}}.$$

The SINR to decode $x_{1}$ at $D_{2}$ is computed as

(7) $$\gamma_{D_{2}\leftarrow x_{1}}=\frac{\varepsilon_{1}P_{RU}{\left|g_{2}\right|}^{2}}{\varepsilon_{2}P_{RU}{\left|g_{2}\right|}^{2}+P_{PS}{\left|h_{p_{2}}\right|}^{2}+N_{0}}.$$

After SIC, the SINR to decode $x_{2}$ is given by

(8) $$\gamma_{D_{2},x_{2}}=\frac{\varepsilon_{2}P_{RU}{\left|g_{2}\right|}^{2}}{P_{PS}{\left|h_{p_{2}}\right|}^{2}+N_{0}}.$$

The dedicated $D2D-Tx_{k}$ with index *k* can be selected to strengthen the $D2D-Tx_{k}$ to RU link as follows:

(9) $$k^{*}=\arg\underset{k=1,\ldots ,K}{\max_{︸}}\left({\left|g_{D_{k}C}\right|}^{2}\right).$$

The CDF and PDF of channel link $D2D-Tx_{k}$ to RU follows

(10) $$F_{{\left|g_{D_{k*}C}\right|}^{2}}\left(z\right)=1-\sum_{k=1}^{K}\left(\begin{array}{c} K\\ k \end{array}\right){\left(-1\right)}^{k-1}\exp\left(-\frac{kz}{\lambda_{DC}}\right),$$

and

(11) $$f_{{\left|g_{D_{k*}C}\right|}^{2}}\left(z\right)=\sum_{k=1}^{K}\left(\begin{array}{c} K\\ k \end{array}\right){\left(-1\right)}^{k-1}\frac{k}{\lambda_{DC}}\exp\left(-\frac{kz}{\lambda_{DC}}\right).$$

in which all links from the group of $D2D-Tx_{k}$ to RU is assumed as equal channel gains $\lambda_{DC}$.

## 3. Outage Probability Analysis in NOMA

It is necessary to evaluate the outage behavior for considered system when the user quality of service (QoS) requirements can be satisfied in the communication system. The outage probability of transmission links over Rayleigh fading channels is examined for different users under two scenarios related to SIC operation.

### 3.1. Outage Probability at User $D_{1}$

According to NOMA scheme, the outage would not occur for the RU’s signal detection related to signal of user $D_{1}$ and also would not happen detect its own information at destination. Such outage performance can be expressed by [38]

(12) $$\begin{array}{cc} P_{D_{1}} & =\mathrm{Pr}\left(\gamma_{D_{k*}C,x_{1}}<\gamma_{1}\cup \gamma_{D_{1},x_{1}}<\gamma_{1}\right)\\ \; & =1-\mathrm{Pr}\left(\gamma_{D_{k*}C,x_{1}}>\gamma_{1},\gamma_{D_{1},x_{1}}>\gamma_{1}\right)\\ \; & =1-\underset{A_{1}}{\underset{︸}{\mathrm{Pr}\left(\gamma_{D_{k*}C,x_{1}}>\gamma_{1}\right)}}\times \underset{A_{2}}{\underset{︸}{\mathrm{Pr}\left(\gamma_{D_{1},x_{1}}>\gamma_{1}\right)}}, \end{array}$$

where it is assumed that $P=P_{BS}=P_{D2D}=P_{PS}$. $\gamma_{i}=2^{2R_{i}}-1$ and $R_{i}$ is the target rate to decode $x_{i},i\in \left\{1,2\right\}$.

**Proposition** **1.** *The closed-form expression of outage probability at $D_{1}$ can be given by*

(13) $$\begin{array}{cc} P_{D_{1}} & =1-\sum_{k=1}^{K}\left(\begin{array}{c} K\\ k \end{array}\right){\left(-1\right)}^{k-1}\frac{P\left(\varepsilon_{1}-\gamma_{1}\varepsilon_{2}\right)\lambda_{DC}}{k\gamma_{1}P\lambda_{pb}+P\left(\varepsilon_{1}-\gamma_{1}\varepsilon_{2}\right)\lambda_{DC}}\exp\left(-\frac{k\gamma_{1}N_{0}}{P\left(\varepsilon_{1}-\gamma_{1}\varepsilon_{2}\right)\lambda_{DC}}\right)\\ \; & \times \frac{P\left(\varepsilon_{1}-\gamma_{1}\varepsilon_{2}\right)\lambda_{1}}{\gamma_{1}P\lambda_{p_{1}}+P\left(\varepsilon_{1}-\gamma_{1}\varepsilon_{2}\right)\lambda_{1}}\exp\left(-\frac{\gamma_{1}N_{0}}{P\left(\varepsilon_{1}-\gamma_{1}\varepsilon_{2}\right)\lambda_{1}}\right). \end{array}$$

**Proof:**  See in Appendix A.

### 3.2. Outage Probability at User $D_{2}$

Regarding performance evaluation at user $D_{2}$, capability needs to be computed to detect $D_{1}$’s information and also can detect its own information during the two slots. In particular, outage probability at user $D_{2}$ can be given as

(14) $$\begin{array}{cc} P_{D_{2}} & =\mathrm{Pr}\left(\gamma_{D_{k*}C,x_{2}}<\gamma_{2}\cup \gamma_{D_{2},x_{2}}<\gamma_{2}\cup \gamma_{D_{2}\leftarrow x_{1}}<\gamma_{2}\right)\\ \; & =1-\mathrm{Pr}\left(\gamma_{D_{k*}C,x_{2}}>\gamma_{2},\gamma_{D_{2},x_{2}}>\gamma_{2},\gamma_{D_{2}\leftarrow x_{1}}>\gamma_{2}\right)\\ \; & =1-\underset{B_{1}}{\underset{︸}{\mathrm{Pr}\left(\gamma_{D_{k*}C,x_{2}}>\gamma_{2}\right)}}\times \underset{B_{2}}{\underset{︸}{\mathrm{Pr}\left(\gamma_{D_{2},x_{2}}>\gamma_{2}\right)}}\times \underset{B_{3}}{\underset{︸}{\mathrm{Pr}\left(\gamma_{D_{2}\leftarrow x_{1}}>\gamma_{2}\right)}}. \end{array}$$

**Proposition** **2.** *The outage performance of $D_{2}$ can be expressed by*

(15) $$\begin{array}{cc} P_{D_{2}} & =1-\sum_{k=1}^{K}\left(\begin{array}{c} K\\ k \end{array}\right){\left(-1\right)}^{k-1}\frac{\varepsilon_{2}P\lambda_{DC}}{k\gamma_{2}P\lambda_{pb}+\varepsilon_{2}P\lambda_{DC}}\exp\left(-\frac{k\gamma_{2}N_{0}}{\varepsilon_{2}P\lambda_{DC}}\right)\\ \; & \times \frac{\varepsilon_{2}P\lambda_{2}}{\gamma_{2}P\lambda_{p_{2}}+\varepsilon_{2}P\lambda_{2}}\exp\left(-\frac{\gamma_{2}N_{0}}{\varepsilon_{2}P\lambda_{2}}\right)\\ \; & \times \frac{P\left(\varepsilon_{1}-\gamma_{2}\varepsilon_{2}\right)\lambda_{2}}{\gamma_{2}P\lambda_{p_{2}}+P\left(\varepsilon_{1}-\gamma_{2}\varepsilon_{2}\right)\lambda_{2}}\exp\left(-\frac{\gamma_{2}N_{0}}{P\left(\varepsilon_{1}-\gamma_{2}\varepsilon_{2}\right)\lambda_{2}}\right). \end{array}$$

**Proof:**  See in Appendix B.

### 3.3. Consideration on Imperfect SIC

On worse case of imperfect SIC at user $D_{2}$, the SINR to decode $x_{2}$ at RU is given by

(16) $$\begin{array}{c} \gamma_{D_{k}C,x_{2}}^{ip}=\frac{\varepsilon_{2}P{\left|g_{D_{k}C}\right|}^{2}}{\varepsilon_{1}P{\left|h_{D_{k}C}\right|}^{2}+P{\left|h_{pb}\right|}^{2}+N_{0}}, \end{array}$$

where $h_{D_{k}C}∼CN\left(0,\tau \lambda_{DCip}\right)$, and τ,0≤τ≤1 is the level of residual interference caused by imperfect SIC.

As imperfect SIC happens at user $D_{2}$, the SINR to decode $x_{2}$ at $D_{2}$ is computed by

(17) $$\begin{array}{c} \gamma_{D_{2},x_{2}}^{ip}=\frac{\varepsilon_{2}P{\left|g_{2}\right|}^{2}}{\varepsilon_{1}P{\left|h_{2}\right|}^{2}+P{\left|h_{p_{2}}\right|}^{2}+N_{0}}, \end{array}$$

where $h_{2}∼CN\left(0,\tau \lambda_{2ip}\right)$.

It need be re-evaluated outage performance of user $D_{2}$ in worse case of imperfect SIC and it is given as

(18) $$\begin{array}{cc} P_{D_{2}}^{ip} & =\mathrm{Pr}\left(\gamma_{D_{k*}C,x_{2}}^{ip}<\gamma_{2}\cup \gamma_{D_{2},x_{2}}^{ip}<\gamma_{2}\cup \gamma_{D_{2}\leftarrow x_{1}}<\gamma_{2}\right)\\ \; & =1-\mathrm{Pr}\left(\gamma_{D_{k*}C,x_{2}}^{ip}>\gamma_{2},\gamma_{D_{2},x_{2}}^{ip}>\gamma_{2},\gamma_{D_{2}\leftarrow x_{1}}>\gamma_{2}\right)\\ \; & =1-\underset{C_{1}}{\underset{︸}{\mathrm{Pr}\left(\gamma_{D_{k*}C,x_{2}}^{ip}>\gamma_{2}\right)}}\times \underset{C_{2}}{\underset{︸}{\mathrm{Pr}\left(\gamma_{D_{2},x_{2}}^{ip}>\gamma_{2}\right)}}\times \mathrm{Pr}\left(\gamma_{D_{2}\leftarrow x_{1}}>\gamma_{2}\right). \end{array}$$

**Proposition** **3.** *The closed-form expression to evaluate outage performance, i.e., $P_{D_{2}}^{ip}$ can be expressed by*

(19) $$\begin{array}{cc} P_{D_{2}}^{ip} & =\sum_{k=1}^{K}\left(\begin{array}{c} K\\ k \end{array}\right)\sum_{t=1}^{K}\left(\begin{array}{c} K\\ t \end{array}\right){\left(-1\right)}^{k+t-2}\exp\left(-\frac{k\gamma_{2}N_{0}}{\varepsilon_{2}P\lambda_{DC}}\right)\\ \; & \times \frac{t\varepsilon_{2}\lambda_{DC}}{k\gamma_{2}\varepsilon_{1}\lambda_{DCip}+t\varepsilon_{2}\lambda_{DC}}\times \frac{\varepsilon_{2}P\lambda_{DC}}{k\gamma_{2}P\lambda_{pb}+\varepsilon_{2}P\lambda_{DC}}\\ \; & \times \frac{\varepsilon_{2}\varepsilon_{2}P\lambda_{2}\lambda_{2}}{\left(\gamma_{2}\varepsilon_{1}\lambda_{2ip}+\varepsilon_{2}\lambda_{2}\right)\left(\gamma_{2}P\lambda_{p_{2}}+\varepsilon_{2}P\lambda_{2}\right)}\exp\left(-\frac{\gamma_{2}N_{0}}{\varepsilon_{2}P\lambda_{2}}\right)\\ \; & \times \frac{P\left(\varepsilon_{1}-\gamma_{2}\varepsilon_{2}\right)\lambda_{2}}{\gamma_{2}P\lambda_{p_{2}}+P\left(\varepsilon_{1}-\gamma_{2}\varepsilon_{2}\right)\lambda_{2}}\exp\left(-\frac{\gamma_{2}N_{0}}{P\left(\varepsilon_{1}-\gamma_{2}\varepsilon_{2}\right)\lambda_{2}}\right). \end{array}$$

**Proof:**  See in Appendix C.

### 3.4. Asymptotic in NOMA

To provide insights in such system, as P→∞ some asymptotic computation of outage evaluations for two users $D_{1}$, $D_{2}$ respectively as

(20) $$\begin{array}{c} P_{D_{1}-high}=1-\sum_{k=1}^{K}\left(\begin{array}{c} K\\ k \end{array}\right){\left(-1\right)}^{k-1}\frac{\left(\varepsilon_{1}-\gamma_{1}\varepsilon_{2}\right)\lambda_{DC}}{k\gamma_{1}\lambda_{pb}+\left(\varepsilon_{1}-\gamma_{1}\varepsilon_{2}\right)\lambda_{DC}}\frac{\left(\varepsilon_{1}-\gamma_{1}\varepsilon_{2}\right)\lambda_{1}}{\gamma_{1}\lambda_{p_{1}}+\left(\varepsilon_{1}-\gamma_{1}\varepsilon_{2}\right)\lambda_{1}}, \end{array}$$

and

(21) $$\begin{array}{c} P_{D_{2}-high}=1-\sum_{k=1}^{K}\left(\begin{array}{c} K\\ k \end{array}\right){\left(-1\right)}^{k-1}\frac{\varepsilon_{2}\lambda_{DC}}{k\gamma_{2}\lambda_{pb}+\varepsilon_{2}\lambda_{DC}}\frac{\varepsilon_{2}\lambda_{2}}{\gamma_{2}\lambda_{p_{2}}+\varepsilon_{2}\lambda_{2}}\frac{\left(\varepsilon_{1}-\gamma_{2}\varepsilon_{2}\right)\lambda_{2}}{\gamma_{2}\lambda_{p_{2}}+\left(\varepsilon_{1}-\gamma_{2}\varepsilon_{2}\right)\lambda_{2}}. \end{array}$$

Similarly, scenario of imperfect SIC leads to corresponding performance degradation for $D_{2}$ and such outage behavior can be rewritten as

(22) $$\begin{array}{cc} P_{D_{2}-asym}^{ip} & =\sum_{k=1}^{K}\left(\begin{array}{c} K\\ k \end{array}\right)\sum_{t=1}^{K}\left(\begin{array}{c} K\\ t \end{array}\right){\left(-1\right)}^{k+t-2}\frac{t\varepsilon_{2}\lambda_{DC}}{k\gamma_{2}\varepsilon_{1}\lambda_{DCip}+t\varepsilon_{2}\lambda_{DC}}\times \frac{\varepsilon_{2}\lambda_{DC}}{k\gamma_{2}\lambda_{pb}+\varepsilon_{2}\lambda_{DC}}\\ \; & \times \frac{\varepsilon_{2}\varepsilon_{2}\lambda_{2}\lambda_{2}}{\left(\gamma_{2}\varepsilon_{1}\lambda_{2ip}+\varepsilon_{2}\lambda_{2}\right)\left(\gamma_{2}\lambda_{p_{2}}+\varepsilon_{2}\lambda_{2}\right)}\frac{\left(\varepsilon_{1}-\gamma_{2}\varepsilon_{2}\right)\lambda_{2}}{\gamma_{2}\lambda_{p_{2}}+\left(\varepsilon_{1}-\gamma_{2}\varepsilon_{2}\right)\lambda_{2}}. \end{array}$$

## 4. Conventional Multiple Access: OMA Mode

The received signal can be computed at the RU in OMA mode as

(23) $$\begin{array}{c} y_{DCi}^{OMA}=\sqrt{P}g_{D_{k}C}x_{i}+\sqrt{P}h_{pb}x_{BS}+\omega_{RU}. \end{array}$$

The SINR to decode $x_{i}$ at link $D2D-Tx_{k}$ to RU is given by

(24) $$\begin{array}{c} \gamma_{D_{k}C,x_{i}}^{OMA}=\frac{P{\left|g_{D_{k}C}\right|}^{2}}{P{\left|h_{pb}\right|}^{2}+N_{0}}. \end{array}$$

In the second phase, RU transmits the signal consisting of the decoded and re-encoded symbols to the secondary destinations. The received signal at $D_{i},i=1,2$ is given by

(25) $$\begin{array}{c} y_{D_{i}}^{OMA}=\sqrt{P}g_{i}x_{i}+\sqrt{P}h_{p_{i}}x_{p}+\omega_{D_{i}}. \end{array}$$

The SINR to decode $x_{1}$ at $D_{1}$ is given by

(26) $$\begin{array}{c} \gamma_{D_{1},x_{1}}^{OMA}=\frac{P{\left|g_{1}\right|}^{2}}{P{\left|h_{p_{1}}\right|}^{2}+N_{0}}. \end{array}$$

The SINR to decode $x_{2}$ at $D_{2}$ is given by

(27) $$\begin{array}{c} \gamma_{D_{2},x_{2}}^{OMA}=\frac{P{\left|g_{2}\right|}^{2}}{P{\left|h_{p_{2}}\right|}^{2}+N_{0}}. \end{array}$$

### 4.1. Outage Performance at User $D_{1}$ in OMA Mode

In such mode, outage performance for the first destination can be rewritten as

(28) $$\begin{array}{cc} P_{D_{1}}^{OMA} & =\mathrm{Pr}\left(\gamma_{D_{k*}C,x_{1}}^{OMA}<\gamma_{1}^{OMA}\cup \gamma_{D_{1},x_{1}}^{OMA}<\gamma_{1}^{OMA}\right)\\ \; & =1-\mathrm{Pr}\left(\gamma_{D_{k*}C,x_{1}}^{OMA}>\gamma_{1}^{OMA},\gamma_{D_{1},x_{1}}^{OMA}>\gamma_{1}^{OMA}\right)\\ \; & =1-\underset{E_{1}}{\underset{︸}{\mathrm{Pr}\left(\gamma_{D_{k*}C,x_{1}}^{OMA}>\gamma_{1}^{OMA}\right)}}\times \underset{E_{2}}{\underset{︸}{\mathrm{Pr}\left(\gamma_{D_{1},x_{1}}^{OMA}>\gamma_{1}^{OMA}\right)}}, \end{array}$$

where $\gamma_{i}^{OMA}=2^{4R_{i}}-1$.

$E_{1}$ is further computed as

(29) $$\begin{array}{cc} E_{1} & =\mathrm{Pr}\left(\frac{P{\left|g_{D_{k*}C}\right|}^{2}}{P{\left|h_{pb}\right|}^{2}+N_{0}}>\gamma_{1}^{OMA}\right)\\ \; & =\mathrm{Pr}\left({\left|g_{D_{k*}C}\right|}^{2}>\frac{\gamma_{1}^{OMA}\left(P{\left|h_{pb}\right|}^{2}+N_{0}\right)}{P}\right)\\ \; & =\int_{0}^{\infty }\left(1-F_{{\left|g_{D_{k*}C}\right|}^{2}}\left(\frac{\gamma_{1}^{OMA}\left(Px+N_{0}\right)}{P}\right)\right)f_{{\left|h_{pb}\right|}^{2}}\left(x\right)dx. \end{array}$$

Then, $E_{1}$ is rewritten as

(30) $$\begin{array}{cc} E_{1} & =\int_{0}^{\infty }\sum_{k=1}^{K}\left(\begin{array}{c} K\\ k \end{array}\right){\left(-1\right)}^{k-1}\exp\left(-\frac{k\gamma_{1}^{OMA}\left(Px+N_{0}\right)}{P\lambda_{DC}}\right)\frac{1}{\lambda_{pb}}\exp\left(-\frac{x}{\lambda_{pb}}\right)dx\\ \; & =\frac{1}{\lambda_{pb}}\sum_{k=1}^{K}\left(\begin{array}{c} K\\ k \end{array}\right){\left(-1\right)}^{k-1}\exp\left(-\frac{k\gamma_{1}^{OMA}N_{0}}{P\lambda_{DC}}\right)\int_{0}^{\infty }\exp\left(-\left(\frac{k\gamma_{1}^{OMA}P}{P\lambda_{DC}}+\frac{1}{\lambda_{pb}}\right)x\right)dx\\ \; & =\sum_{k=1}^{K}\left(\begin{array}{c} K\\ k \end{array}\right){\left(-1\right)}^{k-1}\frac{P\lambda_{DC}}{k\gamma_{1}^{OMA}P\lambda_{pb}+P\lambda_{DC}}\exp\left(-\frac{k\gamma_{1}^{OMA}N_{0}}{P\lambda_{DC}}\right). \end{array}$$

While $E_{2}$ can be expressed by

(31) $$\begin{array}{cc} E_{2} & =\mathrm{Pr}\left(\frac{P{\left|g_{1}\right|}^{2}}{P{\left|h_{p_{1}}\right|}^{2}+N_{0}}>\gamma_{1}^{OMA}\right)\\ \; & =\mathrm{Pr}\left({\left|g_{1}\right|}^{2}>\frac{\gamma_{1}^{OMA}\left(P{\left|h_{p_{1}}\right|}^{2}+N_{0}\right)}{P}\right)\\ \; & =\int_{0}^{\infty }\left(1-F_{{\left|g_{1}\right|}^{2}}\left(\frac{\gamma_{1}^{OMA}\left(Px+N_{0}\right)}{P}\right)\right)f_{{\left|h_{p_{1}}\right|}^{2}}\left(x\right)dx. \end{array}$$

In similar way, $E_{2}$ can be computed by

(32) $$\begin{array}{cc} E_{2} & =\int_{0}^{\infty }\exp\left(-\frac{\gamma_{1}^{OMA}\left(Px+N_{0}\right)}{P\lambda_{1}}\right)\frac{1}{\lambda_{p_{1}}}\exp\left(-\frac{x}{\lambda_{p_{1}}}\right)dx\\ \; & =\frac{1}{\lambda_{p_{1}}}\exp\left(-\frac{\gamma_{1}^{OMA}N_{0}}{P\lambda_{1}}\right)\int_{0}^{\infty }\exp\left(-\left(\frac{\gamma_{1}^{OMA}P}{P\lambda_{1}}+\frac{1}{\lambda_{p_{1}}}\right)x\right)dx\\ \; & =\frac{P\lambda_{1}}{\gamma_{1}^{OMA}P\lambda_{p_{1}}+P\lambda_{1}}\exp\left(-\frac{\gamma_{1}^{OMA}N_{0}}{P\lambda_{1}}\right). \end{array}$$

As a result, $P_{D_{1}}^{OMA}$ can be formulated by

(33) $$\begin{array}{cc} P_{D_{1}}^{OMA} & =1-\sum_{k=1}^{K}\left(\begin{array}{c} K\\ k \end{array}\right){\left(-1\right)}^{k-1}\frac{P\lambda_{DC}}{k\gamma_{1}^{OMA}P\lambda_{pb}+P\lambda_{DC}}\exp\left(-\frac{k\gamma_{1}^{OMA}N_{0}}{P\lambda_{DC}}\right)\\ \; & \times \frac{P\lambda_{1}}{\gamma_{1}^{OMA}P\lambda_{p_{1}}+P\lambda_{1}}\exp\left(-\frac{\gamma_{1}^{OMA}N_{0}}{P\lambda_{1}}\right). \end{array}$$

### 4.2. Outage Performance at User $D_{2}$ in OMA Mode

By considering condition on outage behavior, outage performance can be seen at user $D_{2}$ in OMA mode as

(34) $$\begin{array}{cc} P_{D_{2}}^{OMA} & =\mathrm{Pr}\left(\gamma_{D_{k*}C,x_{2}}^{OMA}<\gamma_{2}^{OMA}\cup \gamma_{D_{2},x_{2}}^{OMA}<\gamma_{2}^{OMA}\right)\\ \; & =1-\mathrm{Pr}\left(\gamma_{D_{k*}C,x_{2}}^{OMA}>\gamma_{2}^{OMA},\gamma_{D_{2},x_{2}}^{OMA}>\gamma_{2}^{OMA}\right)\\ \; & =1-\underset{F_{1}}{\underset{︸}{\mathrm{Pr}\left(\gamma_{D_{k*}C,x_{2}}^{OMA}>\gamma_{2}^{OMA}\right)}}\times \underset{F_{2}}{\underset{︸}{\mathrm{Pr}\left(\gamma_{D_{2},x_{2}}^{OMA}>\gamma_{2}^{OMA}\right)}}. \end{array}$$

In similar computations of $E_{1}$ and $E_{2}$ it can be achieved $F_{1}$ and $F_{2}$, then $P_{D_{2}}^{OMA}$ is given by

(35) $$\begin{array}{cc} P_{D_{2}}^{OMA} & =1-\sum_{k=1}^{K}\left(\begin{array}{c} K\\ k \end{array}\right){\left(-1\right)}^{k-1}\frac{P\lambda_{DC}}{k\gamma_{2}^{OMA}P\lambda_{pb}+P\lambda_{DC}}\exp\left(-\frac{k\gamma_{2}^{OMA}N_{0}}{P\lambda_{DC}}\right)\\ \; & \times \frac{P\lambda_{2}}{\gamma_{2}^{OMA}P\lambda_{p_{2}}+P\lambda_{2}}\exp\left(-\frac{\gamma_{2}^{OMA}N_{0}}{P\lambda_{2}}\right). \end{array}$$

### 4.3. Asymptotic Computation on Outage Probability in OMA Mode

In case of P→∞, lower bound of outage probability for users $D_{1}$, $D_{2}$ are respectively given as

(36) $$\begin{array}{c} P_{D_{1}-high}^{OMA}=1-\sum_{k=1}^{K}\left(\begin{array}{c} K\\ k \end{array}\right){\left(-1\right)}^{k-1}\frac{\lambda_{DC}}{k\gamma_{1}^{OMA}\lambda_{pb}+\lambda_{DC}}\times \frac{\lambda_{1}}{\gamma_{1}^{OMA}\lambda_{p_{1}}+\lambda_{1}}, \end{array}$$

and

(37) $$\begin{array}{c} P_{D_{2}-high}^{OMA}=1-\sum_{k=1}^{K}\left(\begin{array}{c} K\\ k \end{array}\right){\left(-1\right)}^{k-1}\frac{\lambda_{DC}}{k\gamma_{2}^{OMA}\lambda_{pb}+\lambda_{DC}}\times \frac{\lambda_{2}}{\gamma_{2}^{OMA}\lambda_{p_{2}}+\lambda_{2}}. \end{array}$$

**Remark** **1.** *Given the number of selected sources to serve D2D link, improvement can be achieved in terms of outage performance. It is predicted that asymptotic lines mainly depend on channel gains and target rates, regardless of transmit power level. In addition, performance gap among two uses remain in this scenario, and it is confirmed that fairness can be obtained by varying power allocation factors. Thus, there exists a trade-off between the rates of user $D_{1}$ and $D_{2}$ and outage metric which exhibits the fundamental performance of the system.*

## 5. Simulation Results

In this section, to validate the proposed analytical expressions, the simulation results need be considered in terms of the outage probabilities (OP). We use MATLAB to indicate possible evaluations on such system performance. The related expressions are derived in the previous section. Furthermore, the maximal throughputs are analyzed to evaluate the performance of the CR-D2DNOMA system under different channel conditions.

**Figure 2:**
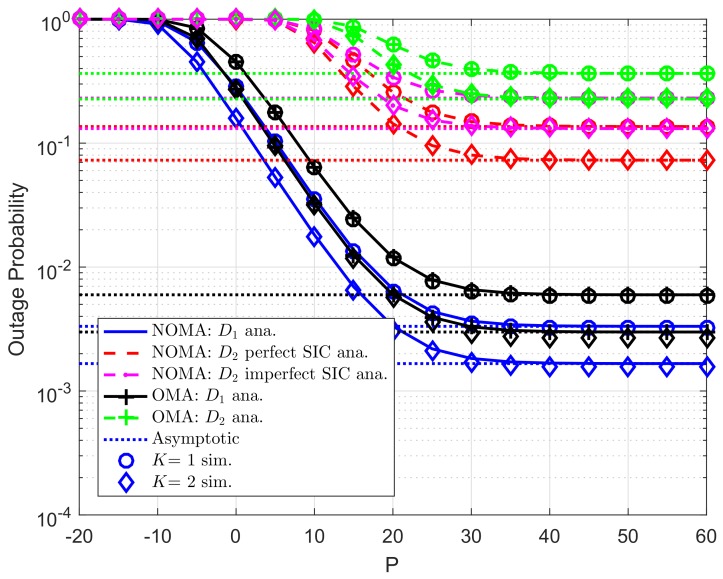
Comparison study on OP of NOMA and OMA versus *P* as changing *K* ($R_{1}=0.5,R_{2}=2$, $N_{0}=1$).

Figure 2 illustrates the outage probability varies under increasing transmit power *P* from −20 (dB) to 60 (dB). More specifically, two scenarios are studied for user $D_{2}$, i.e., imperfect SIC and perfect SIC at the receivers of user $D_{2}$. For these scenarios, we set channel gains of related links as $\lambda_{DC}=\lambda_{1}=\lambda_{2}=10$, $\lambda_{pb}=\lambda_{p_{1}}=\lambda_{p_{2}}=\lambda_{DCip}=\lambda_{2ip}=0.01$. For power allocation factors assigned to each NOMA user, we set $\varepsilon_{1}=0.8$. It can be seen clearly that tight matching simulation and analysis results means the correctness of the analytical outage probability which is verified by simulation results. The asymptotic curves match with exact curves at very high value of *P*. As a result, the correctness of the asymptotic expressions corresponding the considered schemes is also verified. It is shown that user $D_{1}$ achieves the smallest outage probabilities with K=2 in the whole SNR regime. The main reason makes worse outage behavior at user $D_{2}$ is resulted by lower power allocation factor assigned to user $D_{2}$. It is confirmed fairness among two NOMA users can be controlled by such power allocation coefficients. In addition, outage performance in OMA case is worse than that in NOMA for each destination.

**Figure 3:**
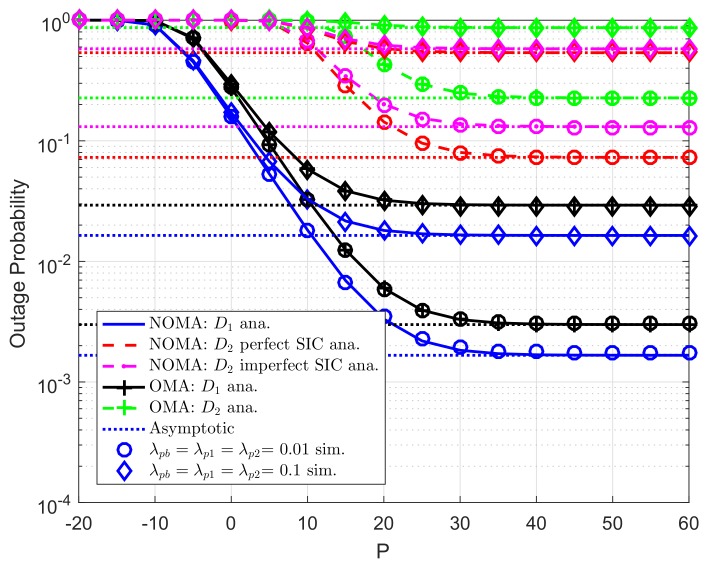
Comparison study on OP of NOMA and OMA versus *P* as changing $\lambda_{pb}=\lambda_{p_{1}}=\lambda_{p_{2}}$ ($\varepsilon_{1}=0.8$, *K*= 3, $N_{0}=1$).

**Figure 4:**
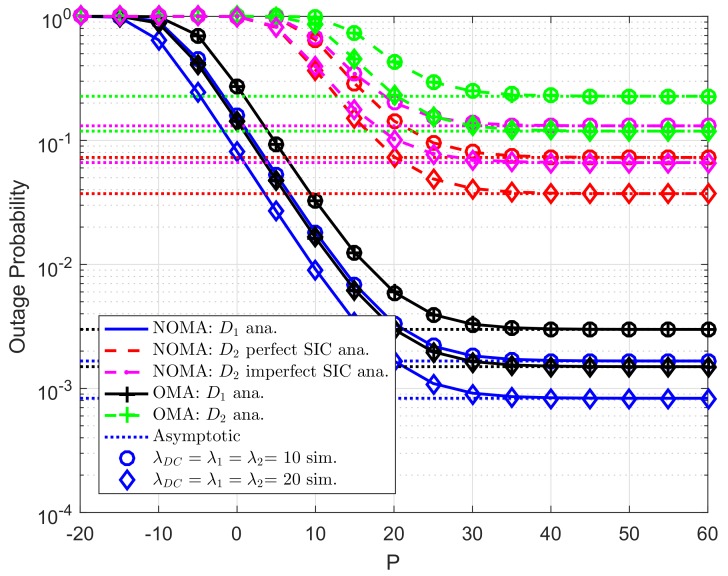
Comparison study on OP of NOMA and OMA versus *P* as changing $\lambda_{DC}=\lambda_{1}=\lambda_{2}$ ($\varepsilon_{1}=0.8$, $R_{1}=0.5,R_{2}=2$, $\lambda_{pb}=\lambda_{p_{1}}=\lambda_{p_{2}}=\lambda_{DCip}=\lambda_{2ip}=0.01$, *K*= 3, $N_{0}=1$).

The analytical and simulated outage probabilities for both OMA and NOMA case at two destinations in CR-D2DNOMA system are shown in Figure 3. The simulated parameters set as $R_{1}=0.5,R_{2}=2$ for target rates, channel gains are $\lambda_{DC}=\lambda_{1}=\lambda_{2}=10$, levels of imperfect SIC are $\lambda_{DCip}=\lambda_{2ip}=0.01$. By increasing transmit power at the user $D2D-Tx_{k}$ or the RU, outage performance will be improved, and such outage probabilities remain stable at high transmit power *P*. Thus, it can be seen that the performance of the CR-D2DNOMA system can be improved significantly by a suitable transmit power design. Similarly, as previous experiment, Monte Carlo simulations are employed to confirm the analytical results. It is evident that the simulation curves match tight with the analytical curves. While Figure 4 indicated how strong channel gains make impacts on outage performance. It can be observed that $\lambda_{DC}=\lambda_{1}=\lambda_{2}=20\left(dB\right)$ provides better performance compared with another.

**Figure 5:**
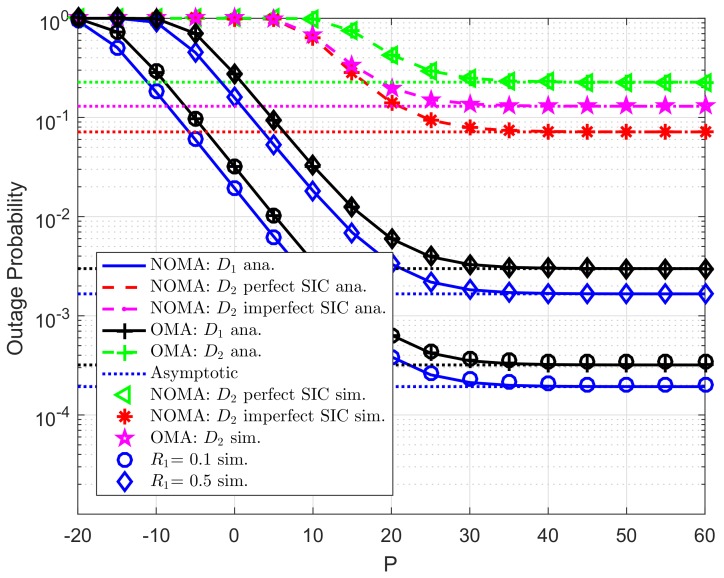
Comparison study on OP of NOMA and OMA versus *P* as changing $R_{1}$ ($\varepsilon_{1}=0.8$, $R_{2}=2$, *K*= 3, $N_{0}=1$).

**Figure 6:**
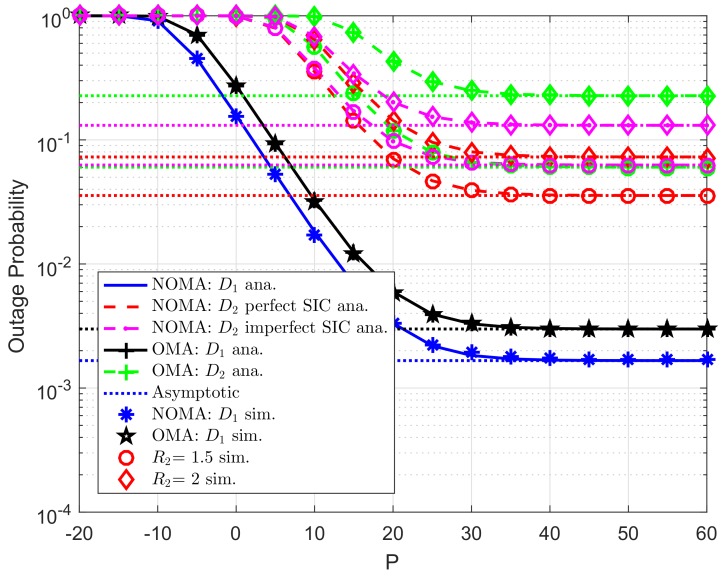
Comparison study on OP of NOMA and OMA versus *P* as changing $R_{2}$ ($\varepsilon_{1}=0.8$, $R_{1}=0.5$, $\lambda_{DC}=\lambda_{1}=\lambda_{2}=10$, $\lambda_{pb}=\lambda_{p_{1}}=\lambda_{p_{2}}=\lambda_{DCip}=\lambda_{2ip}=0.01$, *K*= 3, $N_{0}=1$).

Impact of target rate $R_{1},R_{2}$ on outage behavior can be seen in Figure 5 and Figure 6, respectively. In this experiment, we set $\lambda_{DC}=\lambda_{1}=\lambda_{2}=10$, $\lambda_{pb}=\lambda_{p_{1}}=\lambda_{p_{2}}=\lambda_{DCip}=\lambda_{2ip}=0.01$. It is confirmed that lower required target rate results in improved outage probability. The main reason is that expression of outage probability depends on achievable SINR and the threshold SINR while such SINR term is decided by the target rate. It is worth noting that outage performance gap among two destinations exist different power allocation factors assigned to such users.

**Figure 7:**
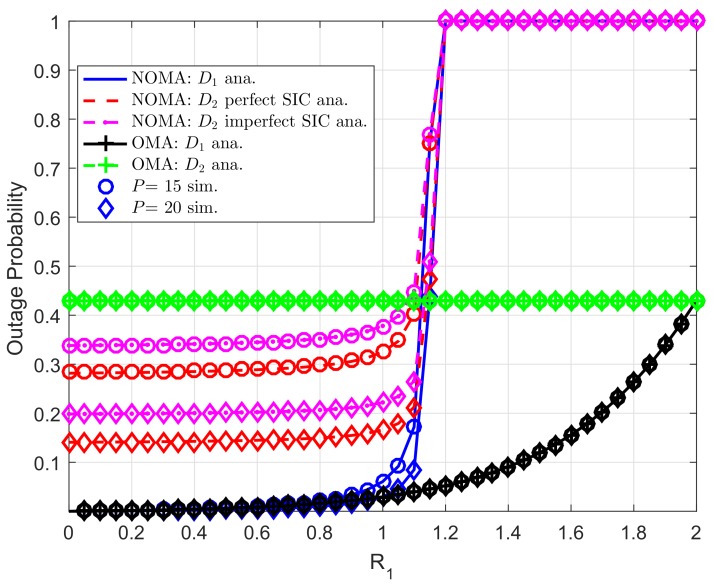
Comparison study on OP of NOMA and OMA versus $R_{1}$ as changing *P* ($\varepsilon_{1}=0.8$, $R_{2}=2$, $\lambda_{DC}=\lambda_{1}=\lambda_{2}=10$, $\lambda_{pb}=\lambda_{p_{1}}=\lambda_{p_{2}}=\lambda_{DCip}=\lambda_{2ip}=0.01$, *K*= 3, $N_{0}=1$).

Figure 7 further examines role of target rate $R_{1}$. At low region of $R_{1}$, outage performance of two destinations are at reasonable values, but outage happens at high regime of $R_{1}$. In this case, interesting point is that OMA-based users achieve outage performance at high region of $R_{1}$.

**Figure 8:**
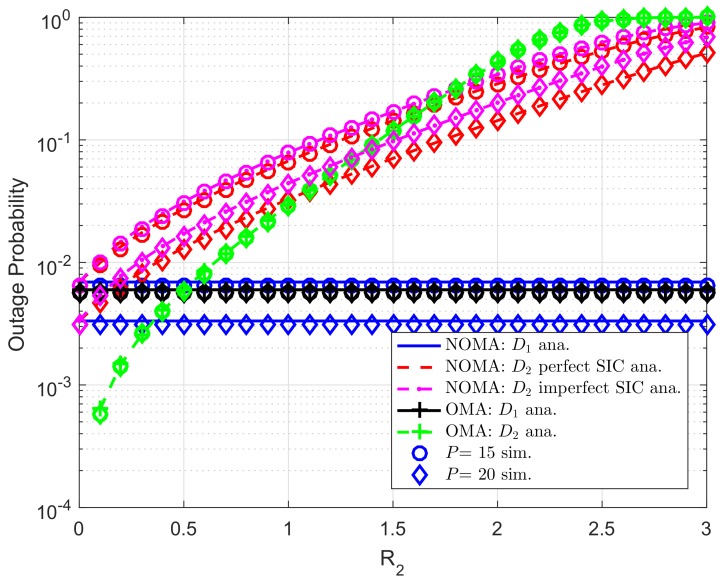
Comparison study on OP of NOMA and OMA versus $R_{2}$ as changing *P* ($\varepsilon_{1}=0.8$, $R_{1}=0.5$, *K*= 3, $N_{0}=1$).

Figure 8 further evaluates outage performance of user $D_{2}$ as increasing $R_{2}$. We set $\lambda_{DC}=\lambda_{1}=\lambda_{2}=10$, $\lambda_{pb}=\lambda_{p_{1}}=\lambda_{p_{2}}=\lambda_{DCip}=\lambda_{2ip}=0.01$. As can be seen from the figure, user $D_{2}$ meets outage event at high target rate $R_{2}$, outage performance of user $D_{1}$ in both NOMA mode and OMA mode does not change in this situation. In this case, higher value of *P* leads to better outage performance for all users.

**Figure 9:**
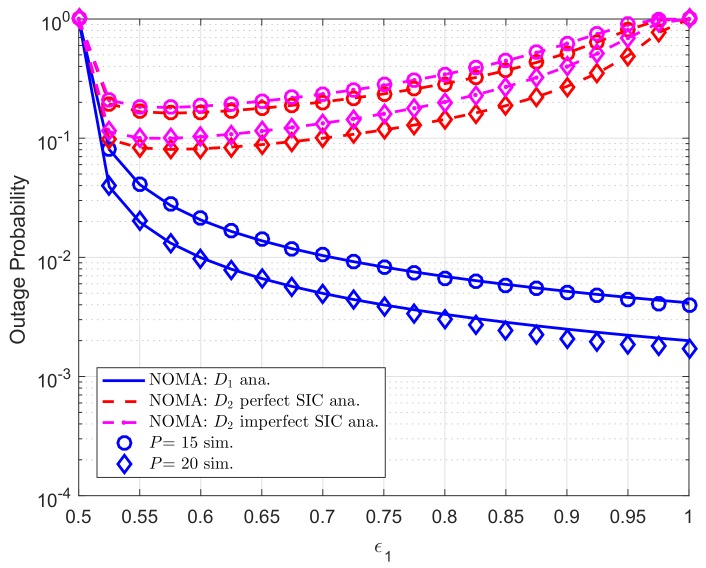
Comparison study on OP of NOMA and OMA versus $\varepsilon_{1}$ as changing *P* ($R_{1}=0.5$, $R_{2}=2$, *K*= 3, $N_{0}=1$).

Figure 9 exhibits optimal outage performance for user $D_{2}$ as increasing $\varepsilon_{1}$ in whole range. We set $\lambda_{DC}=\lambda_{1}=\lambda_{2}=10$, $\lambda_{pb}=\lambda_{p_{1}}=\lambda_{p_{2}}=\lambda_{DCip}=\lambda_{2ip}=0.01$. In particular, best outage performance of user $D_{2}$ can be obtained at approximately $\varepsilon_{1}=0.53$. As can be seen from the figure, user $D_{1}$ meets lowest outage performance at very high value of $\varepsilon_{1}$. It can be explained that power allocation factor $\varepsilon_{1}$ results in varying SINR term and then outage probability of each user varies.

## 6. Conclusions

Cognitive radio and device-to-device, as two important technologies recommended for the forthcoming 5G networks, are studied in this paper to enhance the attainable system performance of secondary network under impact of interference from primary network. More source nodes are selected to serve best transmission on D2D link. Performance gap in terms of outage probability is reported by different power allocation factors assigned to two users. By exploiting numerical simulation, optimal outage performance of User $D_{2}$ can be achieved. This paper indicated that the considered system satisfies acceptable performance at specific target rates and levels of interference channels. Multiple users can be studied in extended work in future.

## Appendix A

**Proof of Proposition** **1.** $A_{1}$ can be computed by

(A1) $$\begin{array}{cc} A_{1} & =\mathrm{Pr}\left(\frac{\varepsilon_{1}P{\left|g_{D_{k*}C}\right|}^{2}}{\varepsilon_{2}P{\left|g_{D_{k*}C}\right|}^{2}+P{\left|h_{pb}\right|}^{2}+N_{0}}>\gamma_{1}\right)\\ \; & =\mathrm{Pr}\left({\left|g_{D_{k*}C}\right|}^{2}>\frac{\gamma_{1}\left(P{\left|h_{pb}\right|}^{2}+N_{0}\right)}{P\left(\varepsilon_{1}-\gamma_{1}\varepsilon_{2}\right)}\right)\\ \; & =\int_{0}^{\infty }\left(1-F_{{\left|g_{D_{k*}C}\right|}^{2}}\left(\frac{\gamma_{1}\left(Px+N_{0}\right)}{P\left(\varepsilon_{1}-\gamma_{1}\varepsilon_{2}\right)}\right)\right)f_{{\left|h_{pb}\right|}^{2}}\left(x\right)dx. \end{array}$$

Applying CDF and PDF, then $A_{1}$ is formulated as

(A2) $$\begin{array}{cc} A_{1} & =\int_{0}^{\infty }\sum_{k=1}^{K}\left(\begin{array}{c} K\\ k \end{array}\right){\left(-1\right)}^{k-1}\exp\left(-\frac{k\gamma_{1}\left(Px+N_{0}\right)}{P\left(\varepsilon_{1}-\gamma_{1}\varepsilon_{2}\right)\lambda_{DC}}\right)\frac{1}{\lambda_{pb}}\exp\left(-\frac{x}{\lambda_{pb}}\right)dx\\ \; & =\sum_{k=1}^{K}\left(\begin{array}{c} K\\ k \end{array}\right){\left(-1\right)}^{k-1}\frac{P\left(\varepsilon_{1}-\gamma_{1}\varepsilon_{2}\right)\lambda_{DC}}{k\gamma_{1}P\lambda_{pb}+P\left(\varepsilon_{1}-\gamma_{1}\varepsilon_{2}\right)\lambda_{DC}}\exp\left(-\frac{k\gamma_{1}N_{0}}{P\left(\varepsilon_{1}-\gamma_{1}\varepsilon_{2}\right)\lambda_{DC}}\right). \end{array}$$

$A_{2}$ is given by

(A3) $$\begin{array}{cc} A_{2} & =\mathrm{Pr}\left(\frac{\varepsilon_{1}P{\left|g_{1}\right|}^{2}}{\varepsilon_{2}P{\left|g_{1}\right|}^{2}+P{\left|h_{p_{1}}\right|}^{2}+N_{0}}>\gamma_{1}\right)\\ \; & =\mathrm{Pr}\left({\left|g_{1}\right|}^{2}>\frac{\gamma_{1}\left(P{\left|h_{p_{1}}\right|}^{2}+N_{0}\right)}{P\left(\varepsilon_{1}-\gamma_{1}\varepsilon_{2}\right)}\right)\\ \; & =\int_{0}^{\infty }\left(1-F_{{\left|g_{1}\right|}^{2}}\left(\frac{\gamma_{1}\left(Px+N_{0}\right)}{P\left(\varepsilon_{1}-\gamma_{1}\varepsilon_{2}\right)}\right)\right)f_{{\left|h_{p_{1}}\right|}^{2}}\left(x\right)dx. \end{array}$$

Similarly, computation of $A_{1}$, by applying CDF and PDF it can be achieved $A_{2}$ as

(A4) $$\begin{array}{cc} A_{2} & =\int_{0}^{\infty }\exp\left(-\frac{\gamma_{1}\left(Px+N_{0}\right)}{P\left(\varepsilon_{1}-\gamma_{1}\varepsilon_{2}\right)\lambda_{1}}\right)\frac{1}{\lambda_{p_{1}}}\exp\left(-\frac{x}{\lambda_{p_{1}}}\right)dx\\ \; & =\frac{P\left(\varepsilon_{1}-\gamma_{1}\varepsilon_{2}\right)\lambda_{1}}{\gamma_{1}P\lambda_{p_{1}}+P\left(\varepsilon_{1}-\gamma_{1}\varepsilon_{2}\right)\lambda_{1}}\exp\left(-\frac{\gamma_{1}N_{0}}{P\left(\varepsilon_{1}-\gamma_{1}\varepsilon_{2}\right)\lambda_{1}}\right). \end{array}$$

Replacing $A_{1}$, $A_{2}$ into (11), it completes the proof. □

## Appendix B

**Proof of Proposition** **2.** It is noted that $B_{1}$ is rewritten as

(A5) $$\begin{array}{cc} B_{1} & =\mathrm{Pr}\left(\frac{\varepsilon_{2}P{\left|g_{D_{k}C}\right|}^{2}}{P{\left|h_{pb}\right|}^{2}+N_{0}}>\gamma_{2}\right)\\ \; & =\mathrm{Pr}\left({\left|g_{D_{k*}C}\right|}^{2}>\frac{\gamma_{2}\left(P{\left|h_{pb}\right|}^{2}+N_{0}\right)}{\varepsilon_{2}P}\right)\\ \; & =\int_{0}^{\infty }\left(1-F_{{\left|g_{D_{k*}C}\right|}^{2}}\left(\frac{\gamma_{2}\left(Px+N_{0}\right)}{\varepsilon_{2}P}\right)\right)f_{{\left|h_{pb}\right|}^{2}}\left(x\right)dx. \end{array}$$

To further compute on $B_{1}$, it can be obtained following result

(A6) $$\begin{array}{cc} B_{1} & =\int_{0}^{\infty }\sum_{k=1}^{K}\left(\begin{array}{c} K\\ k \end{array}\right){\left(-1\right)}^{k-1}\exp\left(-\frac{k\gamma_{2}\left(Px+N_{0}\right)}{\varepsilon_{2}P\lambda_{DC}}\right)\frac{1}{\lambda_{pb}}\exp\left(-\frac{x}{\lambda_{pb}}\right)dx\\ \; & =\sum_{k=1}^{K}\left(\begin{array}{c} K\\ k \end{array}\right){\left(-1\right)}^{k-1}\frac{\varepsilon_{2}P\lambda_{DC}}{k\gamma_{2}P\lambda_{pb}+\varepsilon_{2}P\lambda_{DC}}\exp\left(-\frac{k\gamma_{2}N_{0}}{\varepsilon_{2}P\lambda_{DC}}\right). \end{array}$$

Similarly, $B_{2}$ can be expressed by

(A7) $$\begin{array}{cc} B_{2} & =\mathrm{Pr}\left(\frac{\varepsilon_{2}P{\left|g_{2}\right|}^{2}}{P{\left|h_{p_{2}}\right|}^{2}+N_{0}}>\gamma_{2}\right)\\ \; & =\mathrm{Pr}\left({\left|g_{2}\right|}^{2}>\frac{\gamma_{2}\left(P{\left|h_{p_{2}}\right|}^{2}+N_{0}\right)}{\varepsilon_{2}P}\right)\\ \; & =\int_{0}^{\infty }\left(1-F_{{\left|g_{2}\right|}^{2}}\left(\frac{\gamma_{2}\left(Px+N_{0}\right)}{\varepsilon_{2}P}\right)\right)f_{{\left|h_{p_{2}}\right|}^{2}}\left(x\right)dx. \end{array}$$

By exploiting related CDF and PDF, $B_{2}$ is further computed by

(A8) $$\begin{array}{cc} B_{2} & =\int_{0}^{\infty }\exp\left(-\frac{\gamma_{2}\left(Px+N_{0}\right)}{\varepsilon_{2}P\lambda_{2}}\right)\frac{1}{\lambda_{p_{2}}}\exp\left(-\frac{x}{\lambda_{p_{2}}}\right)dx\\ \; & =\frac{\varepsilon_{2}P\lambda_{2}}{\gamma_{2}P\lambda_{p_{2}}+\varepsilon_{2}P\lambda_{2}}\exp\left(-\frac{\gamma_{2}N_{0}}{\varepsilon_{2}P\lambda_{2}}\right). \end{array}$$

In next steps, $B_{3}$ can be formulated by

(A9) $$\begin{array}{c} B_{3}=\frac{P\left(\varepsilon_{1}-\gamma_{2}\varepsilon_{2}\right)\lambda_{2}}{\gamma_{2}P\lambda_{p_{2}}+P\left(\varepsilon_{1}-\gamma_{2}\varepsilon_{2}\right)\lambda_{2}}\exp\left(-\frac{\gamma_{2}N_{0}}{P\left(\varepsilon_{1}-\gamma_{2}\varepsilon_{2}\right)\lambda_{2}}\right). \end{array}$$

Plugging $B_{1}$, $B_{2}$, $B_{3}$ into (13), the final result can be obtained as in Proposition 2. It is the end of the proof. □

## Appendix C

**Proof of Proposition** **3.** First, $C_{1}$ can be computed as

(A10) $$\begin{array}{cc} C_{1} & =\mathrm{Pr}\left(\frac{\varepsilon_{2}P{\left|g_{D_{k*}C}\right|}^{2}}{\varepsilon_{1}P{\left|h_{D_{k*}C}\right|}^{2}+P{\left|h_{pb}\right|}^{2}+N_{0}}>\gamma_{2}\right)\\ \; & =\mathrm{Pr}\left({\left|g_{D_{k*}C}\right|}^{2}>\frac{\gamma_{2}\left(\varepsilon_{1}P{\left|h_{D_{k*}C}\right|}^{2}+P{\left|h_{pb}\right|}^{2}+N_{0}\right)}{\varepsilon_{2}P}\right)\\ \; & =\int_{0}^{\infty }\int_{0}^{\infty }\left(1-F_{{\left|g_{D_{k*}C}\right|}^{2}}\left(\frac{\gamma_{2}\left(\varepsilon_{1}Px+Py+N_{0}\right)}{\varepsilon_{2}P}\right)\right)f_{{\left|h_{D_{k*}C}\right|}^{2}}\left(x\right)dxf_{{\left|h_{pb}\right|}^{2}}\left(y\right)dy. \end{array}$$

To achieve result, $C_{1}$ need be computed as

(A11) $$\begin{array}{cc} C_{1} & =\int_{0}^{\infty }\int_{0}^{\infty }\sum_{k=1}^{K}\left(\begin{array}{c} K\\ k \end{array}\right){\left(-1\right)}^{k-1}\exp\left(-\frac{k\gamma_{2}\left(\varepsilon_{1}Px+Py+N_{0}\right)}{\varepsilon_{2}P\lambda_{DC}}\right)\\ \; & \times \sum_{t=1}^{K}\left(\begin{array}{c} K\\ t \end{array}\right){\left(-1\right)}^{t-1}\frac{t}{\lambda_{DCip}}\exp\left(-\frac{tx}{\lambda_{DCip}}\right)dx\frac{1}{\lambda_{pb}}\exp\left(-\frac{y}{\lambda_{pb}}\right)dy\\ \; & =\sum_{k=1}^{K}\left(\begin{array}{c} K\\ k \end{array}\right)\sum_{t=1}^{K}\left(\begin{array}{c} K\\ t \end{array}\right){\left(-1\right)}^{k+t-2}\exp\left(-\frac{k\gamma_{2}N_{0}}{\varepsilon_{2}P\lambda_{DC}}\right)\frac{1}{\lambda_{pb}}\frac{t}{\lambda_{DCip}}\\ \; & \int_{0}^{\infty }\exp\left(-\left(\frac{k\gamma_{2}\varepsilon_{1}}{\varepsilon_{2}\lambda_{DC}}+\frac{t}{\lambda_{DCip}}\right)x\right)dx\int_{0}^{\infty }\exp\left(-\left(\frac{k\gamma_{2}P}{\varepsilon_{2}P\lambda_{DC}}+\frac{1}{\lambda_{pb}}\right)y\right)dy\\ \; & =\sum_{k=1}^{K}\left(\begin{array}{c} K\\ k \end{array}\right)\sum_{t=1}^{K}\left(\begin{array}{c} K\\ t \end{array}\right){\left(-1\right)}^{k+t-2}\exp\left(-\frac{k\gamma_{2}N_{0}}{\varepsilon_{2}P\lambda_{DC}}\right)\\ \; & \times \frac{t\varepsilon_{2}\lambda_{DC}}{k\gamma_{2}\varepsilon_{1}\lambda_{DCip}+t\varepsilon_{2}\lambda_{DC}}\times \frac{\varepsilon_{2}P\lambda_{DC}}{k\gamma_{2}P\lambda_{pb}+\varepsilon_{2}P\lambda_{DC}}. \end{array}$$

It can be rewritten $C_{2}$ as below

(A12) $$\begin{array}{cc} C_{2} & =\mathrm{Pr}\left(\frac{\varepsilon_{2}P{\left|g_{2}\right|}^{2}}{\varepsilon_{1}P{\left|h_{2}\right|}^{2}+P{\left|h_{p_{2}}\right|}^{2}+N_{0}}>\gamma_{2}\right)\\ \; & =\mathrm{Pr}\left({\left|g_{2}\right|}^{2}>\frac{\gamma_{2}\left(\varepsilon_{1}P{\left|h_{2}\right|}^{2}+P{\left|h_{p_{2}}\right|}^{2}+N_{0}\right)}{\varepsilon_{2}P}\right)\\ \; & =\int_{0}^{\infty }\int_{0}^{\infty }\left(1-F_{{\left|g_{2}\right|}^{2}}\left(\frac{\gamma_{2}\left(\varepsilon_{1}Px+Py+N_{0}\right)}{\varepsilon_{2}P}\right)\right)f_{{\left|h_{2}\right|}^{2}}\left(x\right)dxf_{{\left|h_{p_{2}}\right|}^{2}}\left(y\right)dy. \end{array}$$

Then, $C_{2}$ is given by

(A13) $$\begin{array}{cc} C_{2} & =\int_{0}^{\infty }\int_{0}^{\infty }\exp\left(-\frac{\gamma_{2}\left(\varepsilon_{1}Px+Py+N_{0}\right)}{\varepsilon_{2}P\lambda_{2}}\right)\frac{1}{\lambda_{2ip}}\exp\left(-\frac{x}{\lambda_{2ip}}\right)dx\frac{1}{\lambda_{p_{2}}}\exp\left(-\frac{y}{\lambda_{p_{2}}}\right)dy\\ \; & =\frac{1}{\lambda_{2ip}}\frac{1}{\lambda_{p_{2}}}\exp\left(-\frac{\gamma_{2}N_{0}}{\varepsilon_{2}P\lambda_{2}}\right)\\ \; & \int_{0}^{\infty }\exp\left(-\left(\frac{\gamma_{2}\varepsilon_{1}}{\varepsilon_{2}\lambda_{2}}+\frac{1}{\lambda_{2ip}}\right)x\right)dx\int_{0}^{\infty }\exp\left(-\left(\frac{\gamma_{2}P}{\varepsilon_{2}P\lambda_{2}}+\frac{1}{\lambda_{p_{2}}}\right)y\right)dy\\ \; & =\frac{\varepsilon_{2}\varepsilon_{2}P\lambda_{2}\lambda_{2}}{\left(\gamma_{2}\varepsilon_{1}\lambda_{2ip}+\varepsilon_{2}\lambda_{2}\right)\left(\gamma_{2}P\lambda_{p_{2}}+\varepsilon_{2}P\lambda_{2}\right)}\exp\left(-\frac{\gamma_{2}N_{0}}{\varepsilon_{2}P\lambda_{2}}\right). \end{array}$$

Plugging $C_{1}$, $C_{2}$, into (17), the final result can be obtained as in Proposition 3. It is the end of the proof.  □
